# Prevalence and root causes of operating room fires in the United States 2014–2024

**DOI:** 10.1186/s13037-025-00441-3

**Published:** 2025-06-02

**Authors:** Monica M. Attia

**Affiliations:** University of California, Davis, Sacramento, CA USA

## Abstract

**Background:**

Operating room fires, though rare, pose serious risks to patient and operator safety. Among the known ignition sources, light-emitting surgical devices—including fiberoptic cables, headlamps, and light boxes—are increasingly recognized contributors. However, the true prevalence and underlying causes remain under-characterized in national surveillance data. This study hypothesized that operator error is a leading cause of light-source-related fires and sought to identify specific device types, procedural timing, and preventable risk factors involved in these adverse events.

**Methods:**

Reports from the U.S. FDA’s MAUDE database were analyzed for light source-related operating room fires from January 1, 2014, to January 1, 2024. Events were categorized by device type, procedural timing, root cause, and resultant injury.

**Results:**

A total of 45 adverse events were analyzed. Most fires were associated with light sources (33.3%), light headlamps (31.1%), and fiberoptic cables (20%). Intraoperative fires comprised the majority (35.6%). Operator error accounted for 37.8% of cases, with common errors including device mishandling (35.2%) and failure to detect damage (17.6%). Only 13.3% required intra-procedural interventions; injuries included one patient burn and two operator injuries.

**Conclusions:**

Most operating room fires involving light sources were linked to modifiable operator errors. These findings underscore the urgent need for preventive strategies—including mandatory training, regular equipment checks, and improved design standards—to reduce intraoperative fire risk and enhance surgical safety.

## Introduction

Operating room fires, though considered rare complications, represent significant safety concerns [1]. According to the Food and Drug Administration’s (FDA) Manufacturer and User Facility Device Experience (MAUDE) database, operating room fires are likely underreported. Light sources have been identified as key ignition mechanisms in the operating room, contributing to these events [2, 3].

The MAUDE database compiles adverse events involving medical devices. Medical device reports (MDRs) include suspected device-associated malfunctions, injuries, and deaths [4]. The database is populated by both mandatory reporters (manufacturers, importers, and device user facilities) and voluntary reporters (healthcare professionals, patients, and consumers) [4]. Thus, the MAUDE database is a passive, postmarket surveillance system that is limited by potentially incomplete, inaccurate, or biased data. Owing to underreporting, data retrieved from the database cannot be used to determine the incidence or prevalence of an event [4]. Nevertheless, the MAUDE database represents a valuable resource for the surveillance of medical devices and provides substantial data that may supplement existing information available through case reports and case series [5].

The purpose of this report was to provide an observational, detailed summary and analysis of the causes, interventions, and complications of light source fires during various procedures. The aim was to raise awareness of the importance of pre- and intraoperative prevention of light source fires and practice advisories to ensure operator and patient safety in operating room procedures. This study could serve as a strong basis for designing a prospective intervention in the future focused on error operating room device safety and error reduction.

## Methods

This study employed a retrospective observational design to evaluate adverse events related to light source fires during operating room procedures in the United States. Data were extracted from the U.S. Food and Drug Administration’s (FDA) Manufacturer and User Facility Device Experience (MAUDE) database over a ten-year period, spanning from January 1, 2014, to January 1, 2024. The MAUDE database is a passive postmarket surveillance system that includes reports submitted by manufacturers, healthcare facilities, and voluntary reporters across the country.

The search strategy involved querying the database for reports categorized under the product classes “light,” “cable,” and “endoscopes,” combined with the problem descriptor “fire.” Reports were included in the analysis if they involved fire events directly linked to medical light source devices, including but not limited to light boxes, headlamps, fiberoptic cables, and associated power sources. Exclusion criteria were applied to remove duplicate entries, cases not involving light source-related fires, reports originating solely from published literature, or those lacking sufficient detail for classification.

The primary outcome of interest was the root cause of the reported fire, categorized as operator error, defective device, both, or undetermined. Secondary outcomes included the type of light source device involved, timing of the event (classified as preoperative, intraoperative, postoperative, or unspecified), interventions required during the procedure, and the presence and type of any resultant injuries to patients or healthcare personnel.

Data were analyzed descriptively. Frequencies and percentages were calculated to characterize the distribution of adverse events across device types, procedural timings, root causes, and outcomes. Cross-tabulations were used to examine associations between device type and other event characteristics, including intervention type and injury outcome. All analyses were observational and exploratory in nature.

This study did not involve human subjects directly and was conducted using publicly available de-identified data. It met the exemption criteria established by the Institutional Review Board of the University of California, Davis.

## Results

A total of 51 MDRs were identified. After six duplicates were excluded, 45 MDRs were included in this study. From these 45 MDRs, 45 adverse events related to sources of fires were identified. Among these 45 adverse events, 36 (80%) occurred during unspecified surgical procedures, three (6.7%) occurred during dental procedures, two (4.4%) occurred during thoracic procedures, one (2.2%) occurred during a urologic procedure, one (2.2%) occurred during an orthopedic procedure, one (2.2%) during a nephrologic procedure, and one (2.2%) during a breast procedure.

When the 45 adverse events by device category were examined, 15 (33.3%) adverse events were related to light sources, 14 (31.1%) were related to light head lamps, 9 (20%) were related to fiber optic cables, and 7 (15.6%) were related to power sources (Table 1). When the number of unspecified procedures (*n* = 18 [40%]) was excluded, the majority of the 45 adverse events occurred intraoperatively (*n* = 16 [35.6%]), followed by preoperatively (*n* = 10 [22.2%]) and postoperatively (*n* = 1, [2.2%]) (Table 1). The majority of the intraoperative adverse events were related to light sources and light head lamps (Table 1). Among the 6 light sources that had intraoperative adverse events, the majority (*n* = 4 [66%]) were related to an unknown component of the light source (Table 1). Among the six light head lamps that had intraoperative adverse events, three (50%) were related to examination lamps, and three (50%) were related to emergency lamps.

**Table 1:** Adverse events by fire source and timing of procedure

Fire source	n (%)	Preoperative, n (%)	Intraoperative, n (%)	Postoperative, n (%)	Unspecified, n (%)
Light box	15 (33.3)	5 (33.3)	6 (40)	0 (0)	4 (26.7)
Unspecified light box	9 (60)	3 (60)	4 (66.7)	0 (0)	2 (40)
Turret	2 (13.3)	1 (20)	0 (0)	0 (0)	1 (25)
Fan or ventilation	2 (13.3)	1 (20)	1 (16.7)	0 (0)	0 (0)
Light guide cable	2 (13.3)	0 (0)	1 (16.7)	0 (0)	1 (25)
Light head lamp	14 (31.1)	2 (14.3)	6 (42.9)	1 (7.1)	5 (35.7)
Fiber Optic cables	9 (20)	3 (33.3)	3 (33.3)	0 (0)	3 (33.3)
Power source	7 (15.6)	0 (0)	1 (14.3)	0 (0)	6 (85.7)
Light wall controller unit	4 (57.1)	0 (0)	1 (100)	0 (0)	3 (50)
Battery	1 (14.3)	0 (0)	0 (0)	0 (0)	1 (16.7)
Power cord	2 (14.3)	0 (0)	0 (0)	0 (0)	2 (16.7)
Total, n (%)	45 (100)	10 (22.2)	16 (35.6)	1 (2.2)	18 (40)

When the root cause of the fire was examined, of the 45 adverse events, most occurred due to operator error (*n* = 17 [37.8%]) when not including the undetermined causes (*n* = 19 [42.2%]) (Table 2). Eight (17.8%) fires occurred due to defective devices, and one (2%) occurred due to both operator error and a defective device (Table 2). Among the 17 factors related to operator error, five (29.4%) were related to a light box, five (29.4%) were related to light head lamps, four (23.5%) were related to a power source, and three (17.6%) were related to fiberoptic cables (Table 2). Among the eight fires related to defective devices as the root cause, three (37.5% related to a power source, two (25%) related to a light box, two (25%) related to a light head lamp, and one (12.5%) related to a fiberoptic cable (Table 2). One fire caused by both operator error and a defective device was related to a fiberoptic light cord cable (Table 2).

**Table 2:** Root cause of light source fires

Fire source	n (%)	Root cause of fire			
		Defective device, n (%)	Operator error, n (%)	Both, n (%)	Undetermined, n (%)
Light box	15 (33.3)	2 (13.3)	5 (33.3)	0 (0)	8 (53.3)
Unknown component	9 (60)	2 (100)	2 (40)	0 (0)	5 (62.5)
Turret	2 (13.3)	0 (0)	1 (20)	0 (0)	1 (12.5)
Fan or ventilation	2 (13.3)	0 (0)	0 (0)	0 (0)	2 (25)
Light guide cable	2 (13.3)	0 (0)	2 (40)	0 (0)	0 (0)
Light head lamp	14 (31.1)	2 (14.3)	5 (35.7)	0 (0)	7 (50)
Examination lamp	10 (71.4)	1 (50)	5 (100)	0 (0)	4 (57.1)
Emergency lamp	4 (28.6)	1 (50)	0 (0)	0 (0)	3 (42.9)
Fiberoptic cables	9 (20)	1 (11.1)	3 (33.3)	1 (11.1)	4 (44.4)
Fiberoptic light cord cables	7 (77.8)	1 (100)	3 (100)	1 (100)	2 (50)
Unknown component	2 (22.2)	0 (0)	0 (0)	0 (0)	2 (50)
Power source	7 (15.6)	3 (42.9)	4 (57.1)	0 (0)	0 (0)
Light wall controller unit	4 (57.1)	0 (0)	4 (100)	0 (0)	0 (0)
Battery	1 (14.3)	1 (33.3)	0 (0)	0 (0)	0 (0)
Power supply	1 (14.3)	1 (33.3)	0 (0)	0 (0)	0 (0)
Power cord	1 (14.3)	1 (33.3)	0 (0)	0 (0)	0 (0)
Total, n (%)	45 (100)	8 (17.8)	17 (37.8)	1 (2.2)	19 (42.2)

Twenty-four (53.3%) of the 45 adverse events did not require intervention, 15 (33.3%) did not specify an intervention, and six (13.3%) required an intervention (Table 3). These six interventions included replacing the device (*n* = 4, [66.7%]) and using the fire extinguisher (*n* = 2, [33.3%]) (Table 4). Replacing a device was a necessary intervention for mainly light head lamps (Table 4). Specifically, of the six replaced devices, two (33.3%) were related to emergency lamps, and one (16.7%) was related to examination lamps (Table 4). A fire extinguisher was used as a necessary intervention only for the light wall controller units (Table 4). Among the 45 adverse events, three (6.7%) resulted in injuries, one related to a patient and two related to the operator (Table 3). The patient's injury was a burn related to a fiberoptic light cord cable caused by improper operator use (Tables 4 and 5). For this adverse event, the intervention was unspecified (Table 3). Two injuries were sustained to an operator via an examination lamp; in particular, the operator’s surgical cap caught fire, and the operator inhaled smoke (Tables 4 and 5). For this particular event, the root cause of the fire was operator error, although the intervention was unspecified (Table 3).

**Table 3:** Interventions and resultant injury by fire source

Category	n (%)	Interventions in procedure			Resultant injury	
		Unspecified, n (%)	Not necessary, n (%)	Necessary, n (%)	Patient, n (%)	Operator, n (%)
Light box	15 (33.3)	5 (33.3)	10 (66.7)	0 (0)	0 (0)	0 (0)
Unknown component	9 (60)	2 (40)	7 (70)	0 (0)	0 (0)	0 (0)
Turret	2 (13.3)	1 (20)	1 (10)	0 (0)	0 (0)	0 (0)
Fan or ventilation	2 (13.3)	2 (40)	0 (0)	0 (0)	0 (0)	0 (0)
Light guide cable	2 (13.3)	0 (0)	2 (20)	0 (0)	0 (0)	0 (0)
Light head lamp	14 (31.1)	4 (29)	7 (50)	3 (21.4)	0 (0)	0 (0)
Examination lamp	10 (71.4)	4 (100)	5 (71.4)	1 (33.3)	0 (0)	2 (20)
Emergency lamp	4 (28.6)	0 (0)	2 (28.6)	2 (66.7)	0 (0)	0 (0)
Fiberoptic cables	9 (20)	4 (44.4)	4 (44.4)	1 (11.1)	0 (0)	0 (0)
Fiberoptic light cord cables	7 (77.8)	3 (75)	3 (75)	1 (100)	1 (14.3)	0 (0)
Unknown component	2 (22.2)	1 (25)	1 (25)	0 (0)	0 (0)	0 (0)
Power source	7 (15.6)	2 (28.6)	3 (42.9)	2 (28.6)	0 (0)	0 (0)
Light wall controller unit	4 (57.1)	1 (50)	1 (33.3)	2 (100)	0 (0)	0 (0)
Battery	1 (14.3)	0 (0)	1 (33.3)	0 (0)	0 (0)	0 (0)
Power supply	1 (14.3)	0 (0)	1 (33.3)	0 (0)	0 (0)	0 (0)
Power cord	1 (14.3)	1 (50)	0 (0)	0 (0)	0 (0)	0 (0)
Total, n (%)	45 (100)	15 (33.3)	24 (53.3)	6 (13.3)	1 (2.2)	2 (4.4)

**Table 4:** Interventions and resultant injuries to the operator and patient by device type

	Light head lamps		Fiberoptic light cord cables, n (%)	Light wall controller unit, n (%)	Total, n (%)
	Examination Lamp, n (%)	Emergency Lamp, n (%)			
Intervention necessary					
Device replaced	1 (33.3)	2 (100)	1 (50)	0 (0)	4 (44.4)
Fire extinguisher	0 (0)	0 (0)	0 (0)	2 (100)	2 (22.2)
Injury type					
Operator burn	1 (33.3)	0 (0)	1 (50)	0 (0)	2 (22.2)
Patient burn	0 (0)	0 (0)	0 (0)	0 (0)	0 (0)
Inhaled smoke by an operator	1 (33.3)	0 (0)	0 (0)	0 (0)	1 (11.1)
Total, n (%)	3 (33.3)	2 (22.2)	2 (22.2)	2 (22.2)	9 (100)

**Table 5:** Root cause of fire by operator error type

Operator error type	n (%)
Tampered or mishandled device	6 (35.2)
Did not conduct proper preventative maintenance	3 (17.6)
Failed to recognize damaged components	3 (17.6)
Used incorrect third-party connectors or bulbs	2 (11.8)
Connected device to improper power supply or circuit breaker	2 (11.8)
Failed to recognize missing components	1 (5.9)
Total, n (%)	17 (100)

Among the 17 adverse events related to operator error (Table 5), the most common reason was related to tampering or mishandling of a device (*n* = 6 [35.2%]), followed by not conducting proper preventative maintenance (*n* = 3 [17.6%]) and failing to recognize damaged components of the device (*n* = 3 [17.6%]), followed by using incorrect third-party connectors or bulbs (*n* = 2 [11.8%]) and connecting the device to an improper power supply or circuit breaker (*n* = 2 [11.8%]), followed by failing to recognize missing components (*n* = 1 [5.9%]).

## Discussion

To the best of my knowledge, this is the first study to explore MAUDE adverse events related to a wide variety of operating room procedure types over 10 years from 2014–2024. This study revealed important findings related to the most implicated devices in the extracted adverse events, root causes of those events, and sequelae. Notably, many of the adverse events appeared to occur in light sources, intraoperatively, and due to operator error.

The present study revealed that the majority of adverse events occurred intraoperatively when the unspecified time of procedure was not considered. This finding is consistent with that of a MAUDE study by Correa et al. that examined malfunction events from 2010–2020 [6]. The authors reported that most adverse events occurring during procedures occurred intraoperatively [6].

Operator error was the most common root cause of fires in this study when the unspecified root causes were not considered. This is commonly supported by findings in the literature. A MAUDE study by Yamasaki et al. examining electrosurgical complications from 2008–2017 revealed that operator errors were more common than device functions [7]. Another MAUDE study by Gopal et al. examining adverse events during urological procedures from 2009–2019 revealed that device malfunctions were most commonly associated with human and operating errors [8]. Patel et al., who likewise examined device malfunctions and complications in urological procedures, reported that, in 61.7% of manufacturer-reviewed cases, 41.3% of operators were the cause of malfunction [9]. An earlier study by Patel et al. examining device-related adverse events during nephrology operations also revealed that the majority of adverse events evaluated were labeled misuse by the operator as the source of malfunction [10].

In the present study, operator errors included tampering or mishandling of devices, not conducting proper preventative maintenance, failing to recognize damaged components, using incorrect third-party connectors or bulbs, connecting devices to improper power supplies or circuit breakers, and failing to recognize missing components. Alemzadeh et al. reported similar common flawed operational practices that contributed to adverse events during operating room procedures: lack of training with specific system features, inadequate troubleshooting of technical problems, inadequate system and maintenance checks before the procedure, incorrect port placements and cable connections, and incorrect manipulation or exchange of instruments [11].

While not identified in this study, Alemzadeh et al. reported that the most common operator error identified was inadequate experience with handling emergencies [11]. This type of operator error was difficult to ascertain from the MAUDE event description in the present study, which raises concerns regarding MAUDE reporting limitations. According to Khalid and Ahmad on the use and applications of MAUDE, reporting is not universally mandated, and guidelines are not entirely standardized [12]. For this reason, many adverse events are likely to be underreported, miscategorized, or not fully characterized [12]. Similarly, Lawal et al. reported that device malfunctions and adverse events are generally underreported despite the availability of reporting guidelines and platforms [13]. With these findings in mind, it is certainly possible that additional unspecified root causes of fires may have been attributed to operator error in the present study, which begs the need for educational intervention aimed at this population.

The resulting injuries were also identified as sequelae of adverse events in this study. The most common type of injury was burns, followed by inhaled smoke. While only two incidents of burn were identified, this is consistent with a MAUDE study by Tremaine and Avran, who identified burns and blisters as the most common adverse events as complications of light source use [14]. Similarly, Yamasaki et al. reported that burn injuries are among the most common patient-related complications of medical devices [5]. Furthermore, operator error was the cause of all the resultant injuries, including the second-degree burn sustained to a patient during the use of a fiberoptic light cord cable and the operator’s surgical cap catching fire in addition to them inhaling smoke during the use of an examination lamp. These injuries are preventable errors that shed further light on the need for operator educational and safety measures related to the use of medical devices.

This study has several notable strengths that enhance its contribution to the literature on operating room safety. The use of a 10-year dataset from the FDA’s MAUDE database provides a robust and extensive sample size that allows for a detailed examination of adverse events related to light source fires across various surgical and procedural settings. By categorizing these events on the basis of device type, root causes, timing, and resultant injuries, this study provides a comprehensive understanding of the factors contributing to such incidents. Furthermore, this study uniquely highlights operator error as the predominant root cause, offering actionable insights for educational interventions and safety advisories. The analysis of specific operator errors, such as device tampering and improper maintenance, provides a practical foundation for targeted quality improvement initiatives.

A limitation of this study is that it does not offer a prospective intervention for light source fires in operating rooms. However, the purpose of this study was to provide an observational nature that provides critical insights for designing and implementing future intervention studies focused on error reduction and safety improvement. Another limitation is that it does not categorize or examine adverse events and outcomes by event date. The data in this study were collected from a 10-year period, and it is not known in the present study whether operator errors, as one example, occurred evenly throughout the time period, early on, or much more recently. Knowledge of this would allow for a better understanding of whether the number of adverse events related to operator error is declining. Another limitation of this study is that it did not align all notable outcomes together, such as which adverse events occurred intraoperatively, which also coincided with which root cause and which intervention, etc. This approach may have elucidated additional information regarding targeted educational interventions, such as at which time of the procedure can the operator benefit from the most education on medical device use. Future studies should develop a more multidimensional approach to categorizing and analyzing MDRs within the limitations of what the MAUDE database provides.

## Conclusion

This 10-year analysis of FDA MAUDE data reveals that a substantial proportion of operating room fires linked to light-emitting surgical devices are preventable and primarily due to operator error. The most frequent errors involved device mishandling and inadequate inspection of equipment before use. These findings highlight a clear opportunity for hospitals and surgical centers to implement targeted quality improvement initiatives, including standardized safety training, preoperative equipment checks, and increased awareness of high-risk device components.

To minimize future incidents, national surgical safety guidelines should incorporate recommendations for routine inspection protocols and mandatory competency training for all operating room personnel. Future prospective studies are needed to assess the effectiveness of such interventions in reducing the incidence of light source-related surgical fires.

## Data Availability

No datasets were generated or analysed during the current study.
